# Editorial

**Published:** 1862-02

**Authors:** 


### Editorial.

### THE AMERICAN MEDICAL ASSOCIATION.

The time is drawing near when some action should be had concerning the Annual Meeting of the American Medical Association. In common with many others, we deemed it advisable that the last annual meeting should not be held. The country was at that time in a state of feverish excitement, and there were few who took a lively interest in anything but current events. Had the meeting been held, we doubt if a respectable number of our medical brethren would have been called together. But the condition of our civil affairs has changed, and this change gives a new tone to the feelings and temper of the people. Business is beginning to resume its former channels, and citizens are returning with increased interest to their former pursuits. The question which we now propose to the medical profession is this: Shall not the American Medical Association hold its annual meeting at Chicago, on the first Tuesday of June next?

So far from the present condition of the country constituting reasonable ground for further postponement, there are several reasons which render a meeting of the Association at this time particularly desirable. The civil contest into which we have been unexpectedly precipitated, develops many new subjects of interest and importance, which it behoves the profession to consider. A host of topics relating to military surgery and hygiene are now, for the first time in our generation, brought home to us, and their careful consideration devolves upon the profession. There will be no dearth of topics which, in the present state of affairs, will spring up in the deliberations of the Association, and which no other organized body of the profession can so appropriately consider. We conceive that the Association owes a duty to the country, the profession, and to itself, which it can only discharge by holding a stated meeting, and remaining in session long enough to deliberate carefully on all the important matters which will come up for consideration. We know that we utter the sentiments of many, when we urge upon the officers of the Association to see to it that the regular meeting in June be seasonably announced.

We copy the above from the *American Medical Times* of February 22d; and fully endorse its sentiments. We think there has been no time in the history of the country, when our Medical Societies, both local and general, could be more useful than at present. We are glad to see that such State Societies as have recently held their annual meetings, have been well attended. For ourselves, we can say to our brethren elsewhere, that we shall greet them in our goodly city with the greatest pleasure. We trust, the proper notices will be issued by the Secretaries and Committee of Arrangements, without delay.

Summer Course of Medical Instruction in this City.—The Medical Faculty of Lind University, will continue a regular course of instruction, to such students as remain in the city, through the summer. The summer course will commence on the first Monday in April, and continue until the first Monday in August. It will embrace one regular *Clinical Lecture* each day, and one familiar lecture and examination on some branch of medical science. Four of the Clinics, each week, will be given in the Hospital, and two in the Dispensary; affording one of the best opportunities for obtaining direct bed-side instruction, in practical medicine, surgery, and the diseases of women and children, that can be found in our country. The hour devoted, each day, to a thorough examination of the class, with such familiar oral instruction as the subject may suggest, is more especially designed to aid the student in a course of systematic and profitable summer reading. This part of the course will embrace instruction in nearly all the branches of medical science. The whole summer course of instruction will be *free* to all who matriculate in the Medical Department of the University.

Illinois State Medical Society.—The Eleventh Regular Annual Meeting of the Illinois State Medical Society will be held at Jacksonville, commencing on the *first Tuesday* in May, 1862. It is very desirable to have a full attendance of delegates and members from all parts of the State.

Chicago, March 1st, 1862. N. S. Davis,
*Permanent Secretary.*

We would call the attention of our readers to the above notice. The meeting of the Society was postponed last Spring, on account of the extreme excitement then existing in reference to the rebellion, that had at that time only just begun. But the first excitement of war has now passed, and there is no good reason why the profession should not resume its customary work, and renew, even more vigorously, all its former efforts for improvement. It is true that many active members of the Society are with our armies; faithfully striving to mitigate the evils of the camp and the battle-field; but this should only stimulate those who have remained at home, to greater exertions to maintain our social organizations, and to push forward the spirit of inquiry and of progress in the profession. Jacksonville is a beautiful city, containing several public institutions of interest, and the local profession will spare no pains to make the meeting both pleasant and profitable. Hence we hope every local Society in the State will be fully represented in the meeting.

The Wounded at Fort Donelson.—In obedience to notice from Gen. Payne, at Cairo, the Chicago Sanitary Commission provided six surgeons from this city for the relief of our gallant soldiers wounded at the battle of Fort Donelson; which number was afterwards swelled to eighteen surgeons and four nurses, by the action of the citizens. These gentlemen, among whom was some of our best surgical talent, went down to the scene of action on Tuesday, the 18th, and remained as long as their services were needed.

From Prof. E. Andrews, M. D., one of the number, we have received the following interesting letter, dated on the *Battle-Field at Fort Donelson*, February 20th, 1862:—

TO THE EDITORS OF THE CHICAGO MEDICAL EXAMINER.

Gentlemen:—The horrible effects of a battle can be but very partially alleviated by the resources of surgical skill. Nevertheless, all that is possible, is here being done for the wounded, both of friend and foe. Fort Donelson occupies a strong position on a hill 150 feet in height, at a bend of the Cumberland River. On the slope of the hill below, are two water batteries of great strength, to sweep the river, but the fort itself, aside from its position, is not strong. It is a hastily built earthwork for infantry, and not an elaborate fortification. It constituted the right wing of the enemy's position. Their rear was protected by the river, and their lines extended along the hills from Fort Donelson three or four miles, terminating at the left wing, on the river again. Along this whole distance, there was a continuous entrenchment. Opposite the right wing, there was a hill covered with rifle-pits, which overlooked and commanded the fort. Near the left wing, there was also an eminence partly cleared of timber, overlooking the intrenchments. These two eminences became the chief point of contest, and the contour of the ground had a marked effect on the kind of surgical injuries produced. In looking over the bodies of the dead on the field, and at the wounded in the hospitals, I am struck with the great number of wounds in the head; and what at first appears strange is the fact, that some bullets traversed from above downward, parallel to the axis of the body. These peculiarities are explained by the fact, that in these hotly contested places, the men availed themselves of the shelter afforded by the rotundity of the hills, by lying flat upon the ground. In this position they loaded and fired a large part of the time, when not advancing or retreating. When raising the head to fire, that part was the only one exposed, and hence the only one at certain periods of the contest which received injury. As the men lay with the head towards the enemy, the peculiar wounds from above occurred, in consequence of this position. The coldness of the weather here prohibits the use of hospital tents as yet, except in extreme necessity, but the houses in the village of Dover, which is within the line, and the steamboats on the river furnish excellent temporary hospitals. The Confederate wounded have been mostly quartered in the houses, while our own men are being rapidly transported by boats to the general hospitals at Paducah and Mound City. This is a most fortunate circumstance, as they are thus removed from the army without the slightest jar or pain to their mangled limbs.

The Government has fitted up the large steamer *City of Memphis* as a floating hospital for this purpose. It is under the superintendence of Assistant Surgeon Turner, formerly of Chicago. In the cabin, which is nearly two hundred feet in length, and in the state-rooms, the patients are crowded together, awaiting their departure down the river. About two hundred are now aboard, reposing on mattresses captured from the enemy.

Accurate statistics of wounds and operations are unattainable at the present time, but the following list showing the location and nature of a large number of injuries, will be of interest:—

| | | | |
|---|---|---|---|
| Wounds of cranium, | 14 | Fractures of hand, | 25 |
| " scalp, | 19 | Flesh wounds, " | 11 |
| " eye, | 4 | Wounds of chest, penetrating cavity, | 10 |
| " jaw, | 4 | " " not " " | 10 |
| " chin, | 2 | " of back, | 5 |
| " tongue, | 1 | " of abdomen, | 7 |
| " ear, | 3 | Fractures of hip, | 7 |
| " mouth, | 4 | Flesh wounds of hip, | 8 |
| " other parts of face, | 10 | Fractures of thigh, | 9 |
| " neck, | 8 | Flesh wounds, " | 37 |
| Fractures of shoulder, | 13 | Fractures of knee, | 2 |
| Flesh wounds of " | 30 | Flesh wounds, " | 7 |
| Fractures of arm, | 16 | Fractures of leg, | 9 |
| Flesh wounds of arm, | 27 | Flesh wounds of leg, | 27 |
| Wounds of elbow, | 4 | Foot fractures, | 4 |
| Fractures of forearm, | 4 | " flesh wounds, | 2 |
| Flesh wounds of " | 4 | Powder burns, | 3 |

The enormous preponderance, here shown, of wounds of the head is very striking, being 61 cases, including the injuries of the face, which is 17 per cent. of the whole list. If, now, we consider that these were all in hospital, while a large portion of those wounded in the head died at once on the field, and are not reckoned, it will be seen that this class of injuries was very numerous. The next most frequent wound was that of the thigh; constituting forty-six of the above list. I am entirely unable to account for the abundance of these cases. The dead generally lay upon their backs, unless their deaths were quite instantaneous. In the latter case they lay as they happened to fall. Hemorrhage on the field was not frequent, still a considerable number of cases occurred, and for days blankets and garments stained with blood, or spotted with brains were visible here and there, where the fight had been the fiercest. I saw even less secondary hemorrhage than primary, quite to my surprise. Very few bayonet wounds were inflicted, which indeed is the usual experience in war. Many cases showed the remarkable tendency of spherical bullets to pursue a tortuous course. This was rarely owing to a glance from the surface of a bone, which is mentioned in the books so prominently as a cause of deflection, but almost always was due to the ball getting into the loose space between the deep and superficial fasciæ. This interfascial space is the favorite track of bullets, because it presents less resistance than either the deeper or more superficial tissues, hence the projectile slides along in it in the most remarkable manner. I saw one case, in which the bullet entered just above the elbow, and gaining this space, went up the whole length of the arm, followed over the curvature of the shoulder, and thence descended to the middle of the back, where it was cut out by an incision through the skin.

The concussion of slight wounds was sometimes remarkable. One man had his ear simply notched by a bullet, yet he fell to the ground and fancied for the time that the side of his head was torn off.

One soldier showed a remarkable mental condition, from a wound of the cranium. The bullet tore across the sagittal suture horizontally, notching both parietal bones, wounding the dura mater, and causing the loss of about half an ounce of brain substance. He has lost entirely the faculty of speech. The voice is not impaired, but he cannot moderate it into words and sentences. On trying to write, he shows the same inability to place language upon paper; yet he seems to retain his general intelligence, and perfectly recognizes his friends, with whom he is very cheerful.

Another man received a spent ball in the lower part of the forehead, just above the nose. The bullet was completely flattened, and on being picked out with the fingers, the skull beneath was found uninjured.

A young man received an oblique shot, entering the brain through the temporal bone. I trepanned him cautiously, removing the depressed bone, and he recovered for a day or two from his comatose condition, but has now relapsed, and will die in a day or two. Many of these brain wounds seem to do well for a few days, but they nearly all die in less than two weeks.

Lieut. Churchill, who I think is from Chicago, is wounded in both thighs. The first one was a flesh wound, received during a *melee*, when some of the enemy were between him and his company. He started back upon the run, when a new shot fractured the opposite thigh. He fell on disputed ground, which was held 20 hours by the enemy, and during that time lay without succor. His feet were slightly frost-bitten; but he is doing well. Amputation of the fractured limb was not thought advisable.

The amputations here present a heavy mortality, in consequence of the shock of the injury which necessitated them.

The hospital steamer, *City of Memphis*, having received again a full cargo of wounded, is ordered to go down the river and discharge at Mound City General Hospital. It is high time; for the unavoidable over-crowding of the past few days has already caused the appearance of traumatic erysipelas among the cabin patients. The hospitals at Mound City are well kept, and very spacious; being capable of accommodating twelve hundred patients. Those at Paducah are also extensive, and well regulated. Several civil surgeons are now here, assisting in the care of the wounded. Most of them are doing good service, but as usual, a few incorrigible blockheads have thrust themselves in where they are not wanted. Cincinnati, St. Louis, Chicago, Springfield, and several other places, are all represented by men who have promptly responded to the call for help. Indeed, the supply is in excess of the demand, and only a portion of the men can be set at work.

Prof. Hollister, of Lind University, is on the Ohio River, superintending the transport of convalescents from the general hospitals to various cities up the river. The military authorities seem to expect more severe battles soon, and are clearing out the convalescents to make room for future wounded.

We have on board the surgeon of the 30th Tennessee regiment—a prisoner. He assists with alacrity in taking care of the patients, and exercises the same impartial care over the wounded of both sides, which is the glory of surgeons in all civilized warfare. The animosity of the field, is not carried to the bedside of the wounded and dying. E. A.

MEDICAL DEPARTMENT OF LIND UNIVERSITY — ANNUAL COMMENCEMENT.—The unexpected delay in the issue of the present number of the EXAMINER, enables us to announce the closing exercises of the third Annual Course of Instruction in this Institution. The exercises of the Public Commencement were held in the Lecture room of the College, on the evening of Marth 4th, 1862. The Lecture room was densely filled with ladies and gentlemen, and, notwithstanding the inclemency of the weather, the occasion was one of much interest. Rev. Dr. PATTERSON opened the exercises by prayer; and the Degree of Doctor of Medicine was conferred by the President of the Faculty, Prof. H. A. JOHNSON, on the following named gentlemen, viz:

Robert Addison, of Illinois.
H. K. Deen, of Indiana.
E. F Dodge, of Wisconsin.
Joseph Haller, of Illinois.
S. Hemenway, of Oregon.
Geo. W. Jones, of Indiana.
A. G. Jones, "
E. H. Neyman, of Iowa.
A. D. Rouse, of Iowa.
G. W. Rohr, of Wisconsin.
E. B. Rockwell, of Illinois.
U. P. Stair, of Wisconsin.
J. F. Taylor, of Missouri.
J. M. Woodworth, of Illinois.
C. H. Bacon, "
O. F. Bartlett, of Wisconsin.

The Honorary Degree was conferred on Dr. F. R. PAYNE, of Marshall, Clark Co., Illinois. The President also announced that the Faculty had awarded the prize for the best Inaugural Thesis, to GEO. W. JONES, of Indiana, and that for the second best, to E. H. NEYMAN, of Iowa. After a brief charge to the Graduates, by the President, the Valedictory Address was delivered by Prof. J. H. HOLLISTER. The address to the graduates upon the "present developments around us, the demands made upon us in this age, as contrasted with ages past, and the particular requisites for successful medical practice," was a well prepared effort, eminently worthy of the head and heart of its author. The advice to new practitioners was sound, calculated to raise the standard of the profession, and enforced with example and anecdote which will have the effect of impressing its lessons indelibly upon the minds of the class to whom it was directed.

After conclusion of the exercises, a pleasant reunion of the faculty, students and invited guests was held at the residence of Dr. N. S. Davis, on Washington street, where refreshments were had, brief addresses were delivered, and general sociability prevailed.

Obituary.—It is with sincere regret that we learn the death of Dr. Joseph N. Graham, of this city. Dr. Graham was an intelligent, active, and highly esteemed member of the profession, and of the community. He began to suffer from the active symptoms of tubercular phthisis, some eighteen months since, and although their progress was partially arrested for a time by travel and change of climate, yet they soon returned, and speedily prostrated their victim. On the morning of the 3d of March, 1862, he sank peacefully to rest; leaving an interesting family, and a large circle of friends to mourn his departure. He was forty years of age. A more extended notice will be prepared hereafter.

Honor to Agassiz.—The London *Lancet* notices that the Copley medal, in the gift of the Royal Society, has been awarded by the Council to the celebrated Agassiz, thus setting the seal to the opinion which has so long prevailed of the merits of the distinguished professor. It is to be regretted that the date at which the Council necessarily make their decision, does not afford time for him to be present to receive the medal in person, at the anniversary meeting of the Society on St. Andrew's day.

---

Propylamin.—Propylamin is a clear, transparent liquid, having a pungent, ammoniacal, alkaline taste and smell. A feeling of causticity is produced when a portion is rubbed between the thumb and finger. It may be derived from a variety of sources,—from ergot, cod-liver oil, bone oil, human urine, etc., but most properly, for medicinal purposes, from herring pickle. When a quantity of old pickle is treated with a strong solution of potassa, a pungent odor like ammonia is evolved, which is propylamin liberated from its combination with an acid in the liquid. The neutral solution must be quickly distilled, and the process continued so long as the fishy odor is observed. The distillate is then saturated with hydrochloric acid, evaporated with much care to a dry crystalline mass, then treated with absolute alcohol, until the propylamin salt is dissolved out. A second careful distillation with hydrate of lime affords a small portion of pure propylamin. I have found that nearly all that should be used for medicinal purposes comes over without the application of heat, or from slight warming. Imperfectly or unskilfully prepared, the remedy will prove worthless, while fresh specimens of *true* propylamin may possess great medicinal value.

The virtues ascribed to propylamin, in the cure of rheumatism and affections of a rheumatic origin, are extraordinary. Dr. Awenarius, of St. Petersburg, has treated (according to a notice translated by Prof. Proctor for the *Journal of Pharmacy*, from Bouchardat's *Repertoire de Pharmacie*) two hundred and fifty patients in the hospital of Kaulinkin, at St. Petersburg, between March, 1854, and June, 1856; and in acute cases the pain and fever *always disappeared the next day.* He regards it "as a true specific for the various affections of rheumatic origin." The diagnosis of these diseases being very often obscure, one can succeed (says M. Awenarius), by the use of propylamin, in bringing to light, in few days, the true nature of the malady. It is stated to have been employed in outside practice with equal success.

Although the claims for the new agent may be, and probably are, extravagant, still should it be found to have, in any measure, control over the specific disease for which it is recommended, it will indeed be a blessing to a suffering class of patients, and therefore merits a trial at the hands of the profession.

The remedy is prescribed in the following manner: R. Propylamin, gtts. xxv.; distilled water, fl. oz. vi.; and, when necessary, add oleo. sacch. peppermint, dr. ij. Dose—a tablespoonful every two hours.—Jas. R. Nichols, *in Boston Med. and Surg. Journal.*

---

Stimulants in Military Hospitals.—Common sense is a large ingredient in the paragraphs we append from *Special Order—No.* 2, issued by Brigade-Surgeon Geo. Suckley, on taking charge of the General Hospital, Cumberland, Md.

"It is desired that, in future, patients in a failing condition will be *stimulated and fed* before they get too low; and it is ORDERED *that no case be treated as hopeless until death has taken place.*

"Stimulus and nourishment in many cases are our only wise medicaments. Food is our best tonic, stimulus gives temporary strength for its digestion. Medical officers practising in the General Hospitals at this Depot, are recommended to combine fluid food with stimulus whenever practicable. Egg-nog, milk-punch, chicken broth, mixed with wine or spirits, are all far better than raw spirits and water, except when a rapid and sudden effect is desired.

"Perhaps there is no better test of a Physician's ability than that afforded by his practice in the administration of stimulants. Nauseating, small spoonfuls frequently repeated, very soon become repulsive. Heroic doses at longer intervals are far better. For example: A small wine-glass of milk-punch administered every fifteen or twenty minutes, scarcely stimulates, and but feebly revives. The patient is incessantly annoyed by the attention and officiousness of his nurse; his rest is broken, and he soon becomes disgusted with the very smell of the mixture. On the contrary, a tumblerful, as near as practicable, given say once in two hours, rouses the whole nervous system; the pulse comes up, a short sleep is gained, the patient is nourished and refreshed.

"In conclusion, it is not only desired (as in paragraph 3) but strongly advised, that the physicians here employed will stimulate and nourish their patients before they run down too low; and not, as is frequently the case, follow an obvious indication only when the suffering patient is at the last gasp—thus justifying the remark once made by an old hospital patient, that he 'never had seen a doctor give a patient brandy unless he was sure to die!'"

Restoration of Bone.—The *Lancet* publishes the following interesting case of restoration of bone, read by M. Demeaux before the Academy of Sciences:—A young soldier had been wounded at Solferino by a musket ball, which had fractured the left superior maxillary. The day after the battle a surgeon extracted the projectile, together with several splinters of bone, in one of which three molars were firmly implanted. On examination, about half of the palatine roofing was found to be devoid of bone, the mucous membrane constituting the only wall of separation between the mouth and nose. In consequence of the want of solidity in the palate, the functions both of articulation and deglutition were impeded. Last summer, Dr. Demaux found the bony plate forming the roof of the mouth quite entire, and the impediment of speech and the inability to swallow had disappeared.

